# Spontaneous thrombosis of a giant common hepatic artery aneurysm—A case report

**DOI:** 10.1002/ccr3.4304

**Published:** 2021-06-10

**Authors:** Athanasios Piachas, Eliza Stavride, Ioannis Lazaridis, Konstantinos Tigkiropoulos

**Affiliations:** ^1^ Department of Surgery Papageorgiou General Hospital Aristotle University of Thessaloniki Thessaloniki Greece; ^2^ Department of Radiology Papageorgiou General Hospital Thessaloniki Greece

**Keywords:** common hepatic artery aneurysm, spontaneous thrombosis, visceral artery aneurysm

## Abstract

Splachnic aneurysms (hepatic artery aneurysms) are a rare entity ranging from atypical symptoms to devastating rupture.

## Graphical abstract

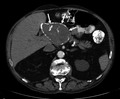

## 1 INTRODUCTION

Hepatic artery aneurysms (HAA) constitute a rare pathology of visceral artery aneurysms with high mortality if left untreated. We present a giant 10 cm aneurysm of the common hepatic artery, spontaneously thrombosed during follow‐up.

**FIGURE 1:**
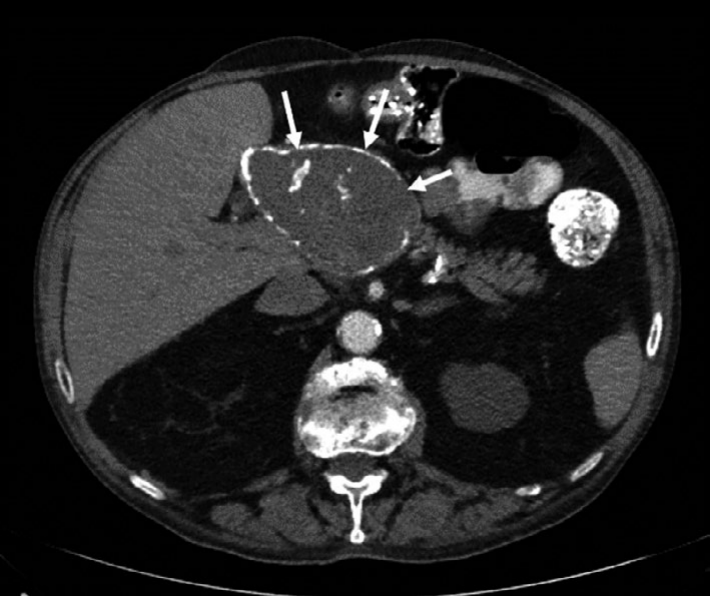
Computed tomography angiography at 24 months depicted spontaneous thrombosis of the common hepatic artery aneurysm (arrows)

Hepatic artery aneurysms (HAA) constitute a rare pathology of visceral artery aneurysms and the second most common visceral aneurysm, following those of the splenic artery. The incidence is estimated to be around 0.002%, while the majority of HAAs are extrahepatic.^1^ Early diagnosis and treatment of HAAs are crucial, due to high mortality if left untreated. We present a case of a patient with a giant 10 cm aneurysm of the common hepatic artery, spontaneously thrombosed during follow‐up. The patient denied any surgical treatment. Computed tomography angiography (CTA) verified thrombosis of the common hepatic artery aneurysm, after a 24 months follow‐up (Figure 1).

## 2 CASE PRESENTATION

**FIGURE 2:**
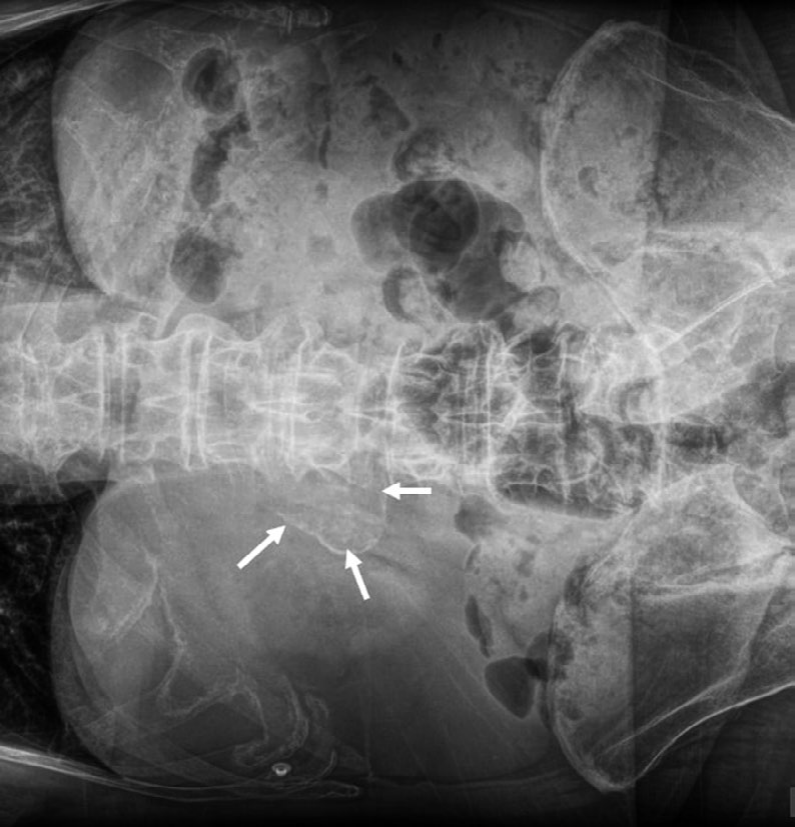
Abdominal radiograph showing an ossified mass projecting at the right paraspinal area (arrows)

**FIGURE 3:**
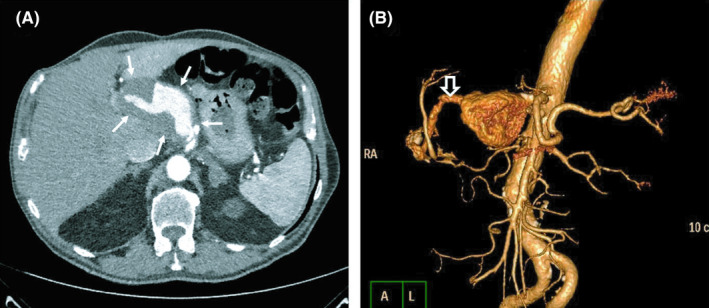
A, Axial and B, 3‐dimensional view of CTA showing a giant common hepatic artery aneurysm. The distal side of the common hepatic artery is shown (empty arrow)

A 61‐year‐old male patient presented to our outpatient surgical ward due to mild pain in the upper epigastric region. His medical history was remarkable for vagotomy due to duodenal ulcer 7 years ago, hypertension, coronary artery disease, and end‐stage renal disease. Initial workup consisted of a plain rardiograph of the abdomen, showing an ossified mass projecting at the right paraspinal area (Figure 2). Subsequent computed tomography angiography (CTA) was performed depicting a 10 cm aneurysm of the common hepatic artery (Figure 3). After a thorough discussion, the patient denied any surgical treatment. The patient was under follow‐up in our outpatient surgical ward, with monitoring his blood pressure in regular basis. After a 24 months follow‐up, CTA verified thrombosis of the common hepatic artery aneurysm (Figure 1).

## 3 DISCUSSION

Hepatic artery aneurysms (HAAs) constitute a rare pathology of a great clinical importance. They are considered the second most common visceral aneurysm, following those of the splenic artery. The incidence of HAAs is estimated to be around 0.002%, while the majority of HAAs are extrahepatic.^1^ The breadth of the clinical manifestations depends on the size of the aneurysm, including epigastric pain, obstructive jaundice, rupture, and death. Huge HAAs (>5 cm) are extremely rare.^2^ Treatment options include open surgery, embolization, or ligation.^2^ However, no formal guidelines regarding visceral artery aneurysms exist.^2^ Spontaneous thrombosis of huge HAA, as in the present case, indicates that careful observation for a giant splanchnic aneurysm should be considered as an alternative choice for patients with multiple comorbidities, or patients with small asymptomatic aneurysms.^1^

## 4 CONCLUSION

Hepatic artery aneurysms are extremely rare. Early diagnosis and treatment of HAAs are crucial, due to high mortality if left untreated. Despite the lack of formal guidelines regarding visceral artery aneurysms, surgery comprises the cornerstone of HAAs treatment. However, in our case, we presented a giant 10 cm aneurysm of the common hepatic artery, spontaneously thrombosed during follow‐up. This case indicates that careful observation for a giant splanchnic aneurysm should be considered as an alternative choice for patients with multiple comorbidities, or patients with small asymptomatic aneurysms.

## CONFLICT OF INTEREST

None declared.

## AUTHOR CONTRIBUTIONS

AP: reviewed the literature and wrote the manuscript. ES: made a contribution to drafting and edited the images. IL: reviewed the literature. KT: reviewed the manuscript.

## ETHICAL APPROVAL

The authors declare that the current manuscript is not published elsewhere. This paper does not appear online, either wholly or in part, as a thesis, working paper series or preprint publication.

## CONSENT STATEMENT

A written consent was obtained from the patient for publication of the case report according to the ethical principles of Declaration of Helsinki.

## Data Availability

All the authors confirm that the data supporting the findings of this paper are available within the article [and/or] its supplementary material.
